# Viral Oncolysis — Can Insights from Measles Be Transferred to Canine Distemper Virus?

**DOI:** 10.3390/v6062340

**Published:** 2014-06-11

**Authors:** Stefanie Lapp, Vanessa M. Pfankuche, Wolfgang Baumgärtner, Christina Puff

**Affiliations:** Department of Pathology, University of Veterinary Medicine Hannover, Bünteweg 17, 30559 Hannover, Germany; E-Mails: stefanie.lapp@tiho-hannover.de (S.L.); vanessa.pfankuche@tiho-hannover.de (V.M.P.); christina.puff@tiho-hannover.de (C.P.)

**Keywords:** canine distemper virus, measles virus, tumor treatment, viral oncolysis

## Abstract

Neoplastic diseases represent one of the most common causes of death among humans and animals. Currently available and applied therapeutic options often remain insufficient and unsatisfactory, therefore new and innovative strategies and approaches are highly needed. Periodically, oncolytic viruses have been in the center of interest since the first anecdotal description of their potential usefulness as an anti-tumor treatment concept. Though first reports referred to an incidental measles virus infection causing tumor regression in a patient suffering from lymphoma several decades ago, no final treatment concept has been developed since then. However, numerous viruses, such as herpes-, adeno- and paramyxoviruses, have been investigated, characterized, and modified with the aim to generate a new anti-cancer treatment option. Among the different viruses, measles virus still represents a highly interesting candidate for such an approach. Numerous different tumors of humans including malignant lymphoma, lung and colorectal adenocarcinoma, mesothelioma, and ovarian cancer, have been studied *in vitro* and *in vivo* as potential targets. Moreover, several concepts using different virus preparations are now in clinical trials in humans and may proceed to a new treatment option. Surprisingly, only few studies have investigated viral oncolysis in veterinary medicine. The close relationship between measles virus (MV) and canine distemper virus (CDV), both are morbilliviruses, and the fact that numerous tumors in dogs exhibit similarities to their human counterpart, indicates that both the virus and species dog represent a highly interesting translational model for future research in viral oncolysis. Several recent studies support such an assumption. It is therefore the aim of the present communication to outline the mechanisms of morbillivirus-mediated oncolysis and to stimulate further research in this potentially expanding field of viral oncolysis in a highly suitable translational animal model for the benefit of humans and dogs.

## 1. Introduction

Neoplastic diseases still represent one of the most common causes of death among humans and dogs [1,2,3]. Therefore neoplasms and their therapy represent an important and emerging field of research in human as well as in veterinary medicine. Despite an abundance of numerous treatment options, most tumor therapies offer only limited increase in life expectations and remain insufficient. In this context the histiocytic sarcoma is exemplarily known as an aggressive tumor with limited response to different conventional therapies including chemo- and radiotherapy [4,5,6]. The relative short survival time of this rare neoplasm in humans and dogs stimulated the need for alternative therapeutic options in both species [5,7,8]. Likewise, situations are found in a multitude of other tumors including frequently occurring human tumors like lung cancer, pancreatic carcinomas, and colorectal cancer [9,10,11]. The need for new therapeutic approaches is in addition emphasized by fact that the complexity of neoplastic diseases requires a tumor type specific therapeutic plan and completely new approaches together with traditional concepts or instead of [12]. 

In veterinary medicine, common treatment options, such as surgery, radiation therapy, chemotherapy, hyperthermia, photodynamic therapy, partially accompanied by complementary alternative methods, often remain palliative [13,14,15,16]. This is reflected by the frequently only marginally improved prognosis after different treatment schemes in frequently occurring and treated malignant canine tumors like malignant lymphoma, hemangiosarcoma, and malignant melanoma [17,18,19]. Similarly, the solitary as well as the disseminated variant of the histiocytic sarcoma of dogs represents another example of an aggressive, difficult to treat neoplasm [4]. This is best illustrated by the fact that a combination of chemo- and radiotherapy only marginally improved the median survival time of affected dogs from 123 to 158 days after diagnosis [4].

**Figure 1:**
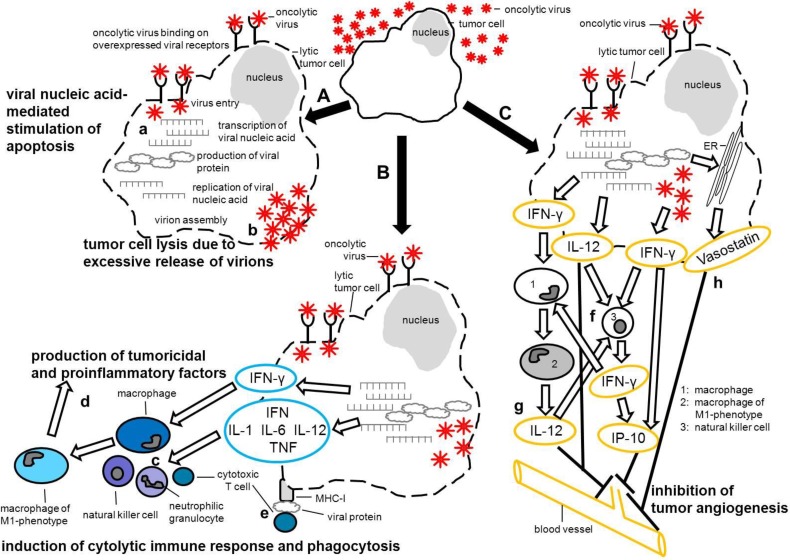
Potential mechanisms leading to tumor cell destruction upon infection with an oncolytic virus. **(A)** Binding to frequently overexpressed virus receptors initiates internalization of the virus into the tumor cell. Viral nucleic acid is released and transcribed which leads to cellular antiviral defense mechanisms such as apoptosis [23,24,25] (**a**). Upon viral gene expression viral proteins are produced exploiting the cellular machinery. Virions are formed by assembly of viral proteins and replication of viral nucleic acids. Tumor cells may subsequently be lysed by massive budding of virions from the cell surface [26] (**b**). **(B)** Pathogen associated molecular pattern (PAMP, viral nucleic acids, viral proteins) stimulate production of antiviral cytokines (IFN, IL-1, IL-6, IL-12, TNF) which in turn lead to attraction of immune cells mediating cytotoxicity and phagocytosis (**c**). IFN-γ polarizes macrophages towards the M1-phenotype [32,33] fostering accumulation of M1‑macrophage derived cytotoxic factors (nitrogen monoxide, inducible nitric oxide synthase, reactive oxygen species) and proinflammatory/angiostatic cytokines (IL-12, TNF) in the tumor microenvironment that may support antitumor treatment [30,31] (**d**). MHC-I mediated presentation of viral proteins activates CD8^+^ cytotoxic T cells, triggering lysis of the oncolytic virus-infected tumor cell (**e**). **(C)** PAMP-enhanced secretion of IFN-γ and IL-12 by the oncolytic virus-infected tumor cell may initiate the complex interplay between IFN-γ, IL-12, NK-cells and IP-10 to eventually limit tumor angiogenesis [34,35,36,37,38] (**f**). IL-12 derived from M1-macrophages may contribute to this process (**g**). Moreover the externalization of the N-terminal fragment of the ER-chaperone protein calreticulin (vasostatin) may be involved in the confinement of tumor vascularization [35,39,40,41] (**h**). **CD**, cluster of differentiation; **ER**, endoplasmic reticulum; **IFN**, interferon; **IFN-γ**, interferon-gamma; **IL**, interleukin; **IP-10**, IFN-γ-inducible protein-10; **MHC**, major histocompatibility complex; **M1**, macrophage 1 phenotype; **TNF**, tumor necrosis factor.

As possible therapeutic alternatives oncolytic viruses have again and again come into focus. Initially, cancer-killing, oncolytic viruses (OVs) were introduced as accidentally found agents predominantly between the 1950s and 1980s [20,21,22]. Though their mode of action with respect to tumor regression remained uncertain, primary (e.g., viral cytolysis, induction of apoptosis) and/or secondary mechanisms (e.g., virucidal immune reactions, alterations within the tumor microenvironment), which both led to tumor cell destruction, were considered (Figure 1A–C). One possible mechanism may include transcription of viral genome and virus release which subsequently triggers cellular defense mechanisms causing tumor cell necrosis and apoptosis. Several studies demonstrated the induction of apoptotic tumor cell death following infection with an oncolytic measles virus [23,24,25]. Furthermore, it has been suggested that, depending on the applied virus strain, one potential mechanism of virus-mediated oncolysis may be based on overwhelming budding and release of virions from the infected tumor cell eventually leading to tumor cell lysis [26] (Figure 1A). Moreover OV-infected tumor cells might be lysed by the innate and adaptive immune responses following attraction of immune cells (Figure 1B). Furthermore, a CD8^+^ T cell response following MHC-I mediated presentation of viral proteins is able to trigger tumor cell lysis (Figure 1B). In addition, tumor associated macrophages (TAM) possess a bivalent role in relation to tumor development. M2-phenotype macrophages are known to enhance tumor progression, whereas M1‑phenotype macrophages play a role in respect to immune surveillance. It is commonly accepted that within a tumor microenvironment macrophages are polarized towards the M2-phenotype [27,28,29]. Therefore, it has been suggested that reprograming M2-tumor associated macrophages towards the tumoricidal and angiostatic M1-phenotype may be beneficial and might stimulate an antitumor response [30,31]. Moreover, antiviral cytokines, such as interferon (IFN)-γ and interleukin (IL)-12, are produced in response to pathogen associated molecular pattern (PAMP) and represent strong initiators of an M1 macrophage response [32,33]. Thus, PAMP-induced IFN-γ and IL-12 production by OV‑infected tumor cells may foster an accumulation of M1-macrophage derived tumoricidal factors and proinflammatory cytokines in the tumor microenvironment (Figure 1B). The same PAMP-induced cytokines may, in addition, initiate the down-stream complex interplay between IFN-γ, IL-12, natural killer cells and ‘angiotoxic’ interferon-γ-inducible protein (IP-10) to eventually depress tumor angiogenesis and thus limit tumor growth and induce tumor regression, respectively. It has been shown that tumor cell derived IL-12 mediated the production of IFN-γ by NK-cells, stimulating in turn the accumulation of IP-10. IP-10-stimulated infiltrating NK-cells have been shown to possess a highly cytolytic capacity upon endothelial cells which resulted in reduction of tumor growth [34,35,36]. Furthermore IP-10 and IL-12 have been shown to inhibit basic fibroblast growth factor, an important proangiogenic protein. IP-10 additionally led to an arrest of capillary tube development [37,38]. Interestingly a canine distemper virus (CDV) infection of Vero cells led to endoplasmic reticulum protein calreticulin fragmentation and translocation of its N-terminal fragment (vasostatin) onto the cell surface [39]. Vasostatin, with and without simultaneous IP-10 treatment, inhibited endothelial cell proliferation and angiogenesis and reduced tumor growth *in vivo* [34,40,41] (Figure 1C).

In order to be safely and effectively employed as anticancer agents, an absent pathogenic effect on non-tumor cells of the host and the ability to selectively infect or replicate in tumor cells is a prerequisite for the use of oncolytic viruses [26,42,43]. Ongoing selection and design of oncolytic viruses, to meet these requirements, has led to potent agents that are thus discriminated into:

1. live-attenuated naturally tumor-selective vaccine strains,
2. selectivity- and efficacy-enhanced, engineered viruses [26,44,45,46,47,48].

**Figure 2:**
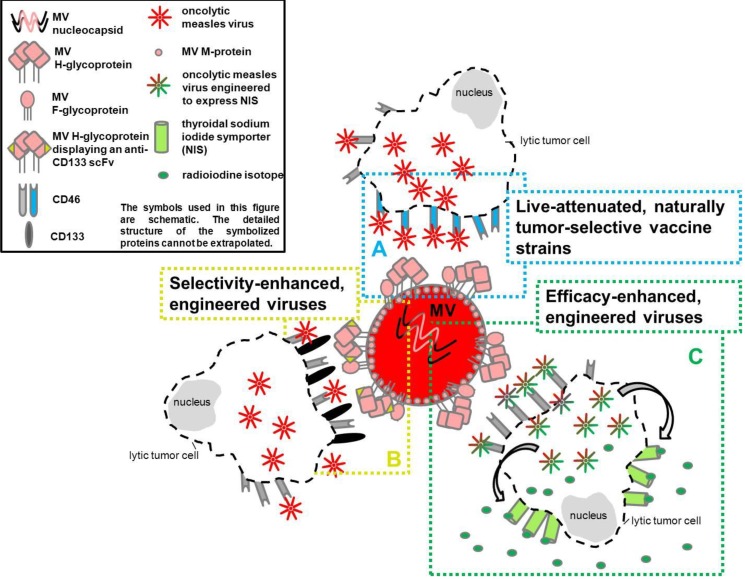
Different types of oncolytic measles virus. (**A**) The H-protein of this live-attenuated naturally tumor selective Edmonston B-strain binds to CD46, a surface receptor frequently overexpressed by tumor cells. Thus, a tumor cell-specific infection with oncolytic MV is facilitated [47,49,50]. (**B**) By genetically fusing a single chain variable fragment directed against CD133 to the H-protein, this measles virus is tumor selectivity-enhanced. It selectively infects CD133-expressing tumor initiating cells, thus, supporting antiproliferative therapies [51]. Tumor initiating cells/cancer stem cells are believed to be a source of recurrent tumor growth after initial antitumor therapy [52]. (**C**) By insertion of cDNA for the human thyroidal sodium iodide symporter (NIS) as an additional transcription unit downstream of the viral hemagglutinin gene, this efficacy-enhanced measles virus leads to expression of NIS by the infected tumor cell [53]. NIS is able to concentrate simultaneously given radioiodine isotopes at the site of tumor implantation, enhancing the radiotherapeutic effect [54]. **CD**, cluster of differentiation; **MV**, measles virus; **MV** **F-glycoprotein**, measles virus fusion glycoprotein; **MV** **H-glycoprotein**, measles virus hemagglutinin glycoprotein; **MV** **M-protein**, measles virus matrix protein; **NIS**, thyroidal sodium iodide symporter; **scFv**, single chain variable fragment.

A schematic overview of different types of oncolytic viruses is given in Figure 2A–C.

Oncolytic viruses comprise wild-type, attenuated or genetically modified virus strains of many different virus families, including *Adeno*-, *Herpes*-, *Paramyxo*-, *Pox*-, and *Reoviridae*, which are able to infect and replicate in tumor cells [46,55,56,57,58]. Particularly, members of the families *Adeno-* and *Herpesviridae* gain attention due to promising results in phase II and phase III clinical trials in humans [48,59,60,61]. One example represents ONYX-015, an oncolytic adenovirus, which was the first oncolytic virus that has been approved for clinical therapy [62]. This virus was designated for treating human head and neck cancer in China [62].

Members of the *Paramyxoviridae* family, namely morbilliviruses like MV and CDV, represent interesting candidates for oncolytic therapy as they display a broad cell tropism [63,64]. Therefore, they might represent an innovative therapy for a wide range of applications and tumor types in both dogs and humans.

## 2. Viral Properties of Measles Virus and Canine Distemper Virus

Measles virus (MV) and canine distemper virus (CDV) both belong to the family *Paramyxoviridae*, genus morbillivirus [63,65]. They are enveloped RNA viruses containing a single stranded negative sense genome [66]. The genome encodes the nucleocapsid (N), phospho- (P), matrix (M), fusion (F), hemagglutinin (H), and large (L) proteins. The two accessory proteins (C and V) are encoded also by the P gene [64,67,68].

### 2.1. Measles Virus

Acute MV infections are clinically associated with a profound immune suppression paving the way for concomitant secondary infectious diseases [69,70]. Additional clinical symptoms include rash and fever [71]. Beside the immunosuppressive effect mediated by MV infection of blood leukocytes with subsequent cell lysis and loss, a viral contact-mediated proliferation inhibition is also described as one of the major mechanisms of lymphoid cell loss and general immune suppression [70,72,73]. Occasionally, namely in subacute and chronic cases, MV can lead to nervous system involvement including primary measles encephalitis, measles inclusion body encephalitis, and subacute sclerosing panencephalitis [71]. 

Present in aerosols and secretions of infected individuals MV can enter the respiratory tract by inhalation followed by a systemic disease [69]. Following respiratory infection, MV can be spread to regional lymph nodes or enter via the tonsils leading to viremia [71]. The infection of lymphatic cells is mediated via CD150 (signaling lymphocyte adhesion molecule, SLAM) [72,74]. Binding of the MV hemagglutinin glycoprotein (H) to CD150 enables attachment of the viral envelope to the designated host cell [74]. CD150 can be found on subsets of lymphocytes, dendritic cells, Langerhans cells and macrophages [75]. Thus, primary cellular targets include immune cells present in the respiratory tract [75]. MV entrance into the cell is facilitated by the F protein mediating pH-independent membrane fusion at the cell surface [66,76]. Alternatively, respiratory tract infection may be facilitated by primary infection of lymphoid cells which transport the virus to the basolateral surface of respiratory epithelial cells [77]. There, MV enters using the basolaterally localized nectin-4 as a cellular receptor [78,79]. Additionally, vaccine and laboratory adapted MV strains can use CD46 as an alternative cellular receptor [64,70,80]. This was achieved in vaccine and laboratory adapted MV strains through amino acid substitutions in the MV hemagglutinin glycoprotein [64,70,80]. 

### 2.2. Canine Distemper Virus

Canine distemper infection associated clinical signs may manifest in a catarrhal or a nervous form or a combination of both [81]. The latter is also termed acute systemic form [81]. The acute infection is associated with a severe and long-lasting lymphocytic depletion and immunosuppression [63]. Furthermore, chronic nervous manifestations, such as old dog encephalitis or chronic demyelinating leukoencephalomyelitis, and various unusual forms, including hard pad disease and enamel hypoplasia of the teeth are described [63,82,83,84]. Overall and in contrast to MV infection central nervous system infection and disease represents a frequent finding in canine distemper [63,85]. In CDV infection, initial virus replication after inhalation takes place in the lymphoid tissue of the respiratory tract [63]. In the lymphoid tissue, virus uptake first occurs in macrophages and monocytes [86]. Von Messling *et al.* (2004) attribute the extensive systemic spread of the virus to an initial infection of circulating B and T cells [87]. Similar to the receptors used by MV, CD46, CD150 (SLAM) and, most recently, nectin 4 have also been described as cellular receptors for CDV [88,89,90,91]. CDV entry into immune cells is, as in MV infection, controlled by the hemagglutinin glycoprotein interacting predominantly with cellular CD150 [92,93]. Recently nectin-4 has been described as an epithelial receptor also for CDV [94], indicating a comparable route of systemic virus distribution as described for MV [77,78,79,94]. Interestingly, in contrast to human measles virus, nectin-4 seems to be involved in CDV neurovirulence [88]. This might be attributed to the broad tissue distribution of nectin-4 in dogs, where this receptor is found, not only on epithelial cells, but also on neurons [88]. In contrast, human nectin-4 is found mainly on epithelial cells, whereas neuronal cells are mainly lacking this receptor [95]. The role of CD46 in CDV infection remains speculative thus far, since this receptor has only been detected in canine lymphoid tumor cells [89]. 

## 3. Measles Virus as an Oncolytic Virus

Measles virus as a potential oncolytic agent was first mentioned in association with anecdotal reports describing regression of hematopoietic malignancies following natural measles virus infection in humans [20,21,22]. As an example, remissions of a Hodgkin lymphoma and long lasting disease free intervals have been reported after accidental measles virus infection in three children [22]. These observations stimulated interest and research to advance oncolytic measles virus therapy. Despite difficulties and draw-backs substantial progress has been achieved in recent years by developing successful *in vitro* and *in vivo* experiments and models, some resulted in phase I clinical trials [50]. For this purpose live attenuated naturally tumor-selective measles vaccine strains as well as selectivity- and efficacy-enhanced, engineered measles virus strains have been generated and employed [46,47,50]. 

### 3.1. Live-Attenuated Naturally Tumor Selective Measles Vaccine Strains

The most consistently used MV strains in oncolytic experimental settings comprise vaccine strains originating from the Edmonston-B (EdMV) strain [47]. EdMV was isolated in 1954 from an 11-year old boy presented with signs of measles virus infection. It became the first measles vaccine strain to be applied to human patients and has been passaged and attenuated *in vitro* multiple times [96].

In naturally occurring measles virus infection of immune cells virus entry is mediated by binding of the viral H protein to CD150 [72,74,90]. In contrast, CD46 represents the predominant entry port for attenuated vaccine strains [80,97,98,99]. Naniche *et al.* (1993) were able to inhibit infection of human CD46-transfected murine cells with EdMV by application of mono- and polyclonal antibodies against CD46. A similar study was performed with hamster cell lines [80]. Santiago *et al.* (2002) and Schneider *et al.* (2002) observed higher association rates of EdMV to CD46 and a higher cell entry efficiency compared to CD150, respectively [98,99]. CD46, present on all nucleated human cells, is a membrane co-factor protein regulating complement mediated lysis of cells [100]. Interestingly, it is frequently overexpressed by tumor cells [101]. Nectin-4, present on respiratory epithelia, can be used by wild-type as well as EdMV for cell entry [78,79]. Due to its overexpression within certain cancer types, nectin-4 has also been implicated as a biomarker for breast, lung and ovarian cancer [101,102,103,104]. Taking into account the usage of CD46 and nectin-4 as viral entry ports and their frequent presence and overexpression on different tumor cells, MV vaccine strains are regarded as potential oncotropic and oncolytic agents (Figure 2A) [47,49,50].

Naturally attenuated vaccine strains and their derivatives have been employed in various cancer models. In a study by Peng *et al.* (2001), EdMV replicated selectively in human myeloma cell lines *in vitro*, displaying syncytial formations and eventual lysis of tumor cells [105]. Infection of myeloma cells before transplantation into immunosuppressed SCID mice resulted in tumor formation only in 1 out of 16 transplanted mice [105]. Myeloma xenografts that received EdMV after establishment of distinct tumors with a diameter of approximately 0.5 cm twice a week regressed completely after seven intratumoral injections with 10^7^ plaque forming units (pfu) [105]. Histopathology and *in situ* hybridization revealed multinucleated syncytia containing abundant EdMV-RNA [105]. Furthermore distant, intravenous delivery of the virus resulted in partial regression of myeloma xenografts with EdMV-RNA present in harvested tumor samples [105].

A comparable study by Grote *et al.* (2001) employed two human B-cell lymphoma cell lines (Raji and DoHH2 cells) [106]. *In vitro* the presence of CD46 was confirmed in both cell lines by flow cytometry [106]. Furthermore, the ability of EdMV and EdMVlacZ (a MV genetically modified by the addition of a β-galactosidase reporter gene) to lytically infect Raji- and DoHH2-cells was demonstrated *in vitro* [106]. Interestingly, preinfection of Raji and DoHH2 cells with EdMV prevented tumor growth after transplantation into SCID mice [106]. Established, non-infected tumors of these cell lines showed a delayed growth after intravenous application of EdMV or EdMVlacZ and a retarded progression or complete regression after intratumoral infection with EdMV or EdMVlacZ [106]. In this study, 10 daily doses of 10^5^ pfu of MVlacZ injected intratumorally in large DoHH2 or Raji tumors (median volume of 0.87 cm^3^) led to tumor stasis or complete regression in 1/5 and 3/7 animals, respectively. In contrast untreated control groups and tumors injected with UV-inactivated virus displayed a progressive growth [106]. In this context, Grote *et al.* [106] demonstrated that smaller Raji tumors (volume < 0.4 cm³) respond to intratumoral MV injections more efficiently than large tumors (volume > 0.4 cm³). A single, intratumoral injection of 10^6^ pfu into DoHH2 tumors lacked a therapeutic benefit [106]. The enhanced response of smaller tumors to oncolytic virus therapy was attributed to physical limitations of virus propagation [106]. Interestingly, the presence of neutralizing anti-MV antibodies did not compromise the antitumor efficacy of intratumoral virus injections [106]. Independently of application routes treated tumors appear histologically similar to controls with the exception of occasional multinucleated syncytia in MV treated tumors [106].

As current research is focusing mainly on engineered and transgene-expressing MV strains to enhance safety, selectivity, and efficacy, few studies use the attenuated, non-modified EdMV strain any longer. 

However, recently an *in vitro*, as well as a xenotransplantation, study on human lung (A549 cells) and colon adenocarcinomas (Caco-2 cells) using the attenuated Schwarz strain of MV, derived from EdMV, was performed [107]. *In vitro*, this MV strain leads to syncytia formation and cell death of Caco-2 and A549 cells. In xenotransplantation studies, Caco-2 adenocarcinomas with a volume of 150–200 mm³ revealed a growth arrest after a single intratumoral injection of the Schwarz MV strain (1.5 × 10^7^ TCID_50_; tissue culture infective dose 50) [107]. In contrast, regression of A549 lung adenocarcinoma xenografts with a volume of 100 mm³ was only achieved after multiple injections (0 d, 22 d, 28 d and 35 d; 1.5 × 10^7^ TCID_50_) [107]. The authors discuss the notably enhanced activation of caspase 3 and subsequent induction of apoptosis as a cause of the antitumor effect seen after Schwarz MV infection [107].

As many virotherapeutics still exhibit only limited efficacy, emphasis has been paid to questions referring to oncoimmunology [108]. To elucidate immune-mediated mechanisms in oncolysis, MV represents a prime candidate for further studies.

An *in vitro* lytic infection of human mesothelioma and melanoma cells after infection with the MV Schwarz and Edmonston strain, respectively was observed [109,110]. Interestingly, co-cultivation of these tumor cell lines with dendritic cells (DC) after MV infection lead to spontaneous maturation of DCs after uptake of MV-infected cellular remnants. This was substantiated by an increased expression of major histocompatibility complex, costimulatory molecules, and an enhanced production of proinflammatory cytokines in DCs [110]. The matured DCs were able to prime T-cells and triggered the proliferation of tumorspecific CD8^+^ T-cells [109,110,111]. In this context, danger associated molecular pattern (DAMP), heat shock proteins, inflammatory cytokines, and pathogen associated molecular pattern (PAMP) released by MV-infected tumor cells induce maturation of myeloid DC (mDC) and plasmacytoid DC (pDC), respectively [111]. Following phagocytosis of specific tumor associated antigens (TAA) by maturing myeloid DCs, these cells gain the ability to cross-present TAA to CD8^+^ T-cells [111]. Plasmacytoid DCs were shown to produce large amounts of IFN-α in response to contact with MV-infected tumor cells (melanoma, mesothelioma, lung adenocarcinoma) possibly enhancing an antitumor immune response [109,110,111,112].

Another naturally occurring mechanism for tumor selectivity of measles virus vaccine strains results from an often defect interferon pathway in neoplastic cells. Berchtold *et al.* (2013) demonstrated that sarcoma cell lines susceptible for measles vaccine virus infection display a weaker, delayed or lacking expression of the virus-recognition receptor retinoic inducible gene-1 (RIG-1), the interferon-induced protein with tetratricopeptide repeats 1 (IFIT1) and a delayed or transient phosphorylation of Stat1 compared to measles vaccine virus resistant sarcoma cell lines. These data suggest that an antiviral innate immune response restricts viral replication limiting the possible success of viral oncolysis in such tumors [113].

To further enhance tumor cell specificity and the efficacy of infection, numerous modifications to the measles virus have been performed and led to the development and investigation of selectivity- and efficacy-enhanced, engineered MV strains.

### 3.2. Selectivity- and Efficacy-Enhanced, Engineered Measles Virus Strains

Many different approaches including an improved virus spread and safety by selective activation of the virus by tumor cells or by modulating the immune system have been performed by genetic engineering [50,114]. These modified viruses are usually based on the EdMV strain and its derivatives [47].

#### 3.2.1. Oncolytic MV-Targeting

##### 3.2.1.1. Receptor-Targeted MV

The unmodified live attenuated EdMV strain has potent antitumor activity but lacks tumor specificity due to its native receptor-specificity to CD46 and SLAM [115]. 

First approaches to modify MV receptor-targeting were published in 2000 [116]. Recombinant EdMV displaying epidermal growth factor (EGF) or the insulin-like growth factor 1 (IGF1) were able to enter CD46-negative rodent cells expressing human EGF or IGF1 receptor. This was the first step to produce viruses which are able to enter cells of choice and interest [116].

Another way to retarget measles virus has been generated by including a single chain antibody directed against tumor-associated carcinoembryonic antigen (CEA) within the viral hemagglutinin [117]. This antigen is commonly overexpressed on the surface of many tumor cells, including colorectal, gastric, lung, pancreatic, and breast carcinoma cells [118]. However, it is also expressed on some normal epithelial cells [119]. Therefore, this approach provides new opportunities for a targeted tumor therapy. 

Based on *in vitro* studies analyzing CD46 and/or CD150 receptor blind MV viruses [120], Nakamura *et al.* (2005) succeeded in full receptor retargeting in 2005 [115]. EdMV efficiently entered cells through tumorselective CD38, epidermal growth factor receptor (EGFR) or EGFR mutant vIII (EGFRvIII) *in vitro* and *in vivo* by lacking CD46 and SLAM specificity.

Commonly used genetic modifications target cell tropism, as exemplarily shown with a myeloma specific MV [121]. This cell type specificity was achieved by inserting a single chain antibody against CD38, a myeloma cell marker, into MV [121]. The aim of the study was to switch the natural MV receptor selectivity to the new specificity domain on target tumor cells to create a tropism expanded MV. *In vitro* experiments using Chinese hamster ovary cells (CHO) expressing CD38 revealed an enhanced susceptibility to the modified MV in contrast to the non-modified MV vaccine strain [121]. Transplantation of these CD38 expressing cells admixed with the modified MV (MV-CD38) in immunodeficient mice revealed an extended cumulative survival time compared to control animals [121].

The replacement of the MV H glycoprotein by an H glycoprotein-single chain anti-CD20 fusion protein depicts a similar approach [122]. CD20 represents a lymphocyte receptor, often expressed in human non-Hodgkin lymphomas [123]. Therefore, the tropism expanded engineered MV, possessing the H glycoprotein-anti-CD20 fusion protein, represents an ideal tool for the treatment of this type of neoplasm [122,124]. *In vitro*, the modified virus replicates in CHO cells expressing human CD20 but not in CHO cells without CD20 expression [122]. In contrast, no substantial differences in the growth characteristics of non-modified attenuated MV and modified MV in HT1080 cells, a human fibrosarcoma cell line, was observed *in vitro*, regardless of a CD20 expression of the tumor cells [122]. A CD20-expressing HT1080 cell xenograft model in immunocompromised mice revealed a diminished progression of tumor growth after systemic administration of the anti-CD20-displaying measles virus (MVHαCD20), whereas this growth retardation was less pronounced in control groups, treated with non-modified MV [122].

Another novel strategy targets CD133 positive cancer-initiating cells using an engineered MV (MV-CD133) [51] (Figure 2B). An *in vivo* model with non-obese diabetic/severe combined immunodeficient (NOD/SCID) mice, which received a subcutaneous xenotransplantation of hepatocellular carcinoma cells (HuH7 cells), revealed a prolonged survival time in intratumorally MV‑CD133 treated animals (1 × 10^6^ TCID_50_ on four consecutive days when tumors reached a diameter of 0.5 cm) compared to controls [51].

##### 3.2.1.2. Protease-Targeted MV

Another promising approach targeted the activation of oncolytic viruses by tumor cell-secreted proteases, enhancing the safety of oncolytic virotherapy in 2006 [125]. One example of such a virus is depicted by a recombinant MV, whose modified envelope fusion protein (F) can be specifically processed and activated by matrix metalloproteinases (MMPs) [125]. The latter are frequently secreted by tumor cells for extracellular matrix degradation [126].

The MV F protein requires activation by proteolytic enzymes, normally achieved by the ubiquitous trans-Golgi protease furin [127]. The replacement of furin-mediated activation of the MV F protein by MMP-2-mediated activation restricts virus spread to MMP secreting cells leading to an enhanced safety profile of the virus and a more tumor cell type specific targeting [125]. This has been proven by an intracerebral inoculation of both, furin and MMP sensitive, viruses in MV-susceptible mice. In this experiment furin-activated virus leads to lethal encephalitis, whereas the MMP-activated virus did not result in a lethal disease, indicating that the modified virus is not pathogenic [125]. However, growth retardation of HT1080 fibrosarcoma xenografts was similar in both MV strains in nude mice [125]. The same modified virus was used to demonstrate the destruction of MMP secreting tumor cells in precision-cut liver slices from human hepatic neoplasms [128]. Moreover, the latter study showed a decreased viral spread in the adjacent unaltered hepatocytes in cell culture experiments [128]. 

##### 3.2.1.3. miRNA-Targeted MV

Recent studies have demonstrated another approach to control the cell or tissue tropism of oncolytic viruses. Therefore, tissue-specific microRNAs (miRNAs), which are known for their regulatory roles in cell proliferation, differentiation, and apoptosis, as well as in tumorigenesis, and their modulation have been applied [129,130,131,132,133]. Leber *et al.* (2011) demonstrated that this strategy can also be assigned to MV. MicroRNA-sensitive MV exhibits potent antitumor activity in glioblastoma cells and subcutaneous xenografts in NOD/SCID mice. The latter model comprises subcutaneous transplantation of 2 × 10^6^ U87 glioblastoma cells, which were injected intratumorally five times with 8 × 10^5^ infectious units of MV-EGFP^miR7^ on five consecutive days when the neoplasms reached a volume of ~ 75 µL [134]. 

#### 3.2.2. Oncolytic MV-Monitoring

Unfortunately, virus tracking and monitoring of virus replication within the tumor/recipient remains difficult using conventional non-invasive methods [135,136,137]. An interesting way to solve this problem is to trace inert soluble marker peptides, for example soluble human CEA, expressed by oncolytic viruses [137]. For this purpose, inert, non-immunogenic, non-functional, and measurable marker peptide-expressing MVs were generated, which do not interfere with virus replication. The concentration of the marker peptides can be determined in serum samples of treated individuals and kinetics or correlations with the therapeutic outcome can be performed [137].

Different *in vitro* and *in vivo* studies using such a replication competent CEA-expressing MV (MV‑CEA) in human epithelial ovarian cancer and glioblastoma multiforme revealed an oncolytic activity with little cytotoxicity to normal cells [137,138]. Direct administration of MV-CEA (five doses of 10^7^ pfu/dose in two weeks) in subcutaneous human ovarian carcinoma xenografts (SKOV3ip.1 cells) with a diameter of approximately 0.5 cm in athymic mice induced complete regression in 80% of the tumors [137]. Following intraperitoneal administration of this virus (16 doses of 10^7^ pfu/dose delivered over a six-week period) in intraperitoneal SKOV3ip.1 xenografts the median survival time of mice was extended from 30 d, in the control group, to >80 d in treated animals [137].

#### 3.2.3. Efficacy-Enhanced/‘Armed’ MV (Oncolytic MV-‘Arming’)

In a medulloblastoma xenotransplantation study in athymic nude mice treatment with an ‘armed’ measles virus with a human thyroidal sodium iodide symporter (MV-NIS; 2 × 10^5^ pfu) led to a prolonged survival time, which could further be increased by simultaneous application of radioiodine (^131^I; 37MBq) [54] (Figure 2C). Interestingly, an additional insertion of the IFN-β gene into MV-NIS resulted in an accumulated immune cell infiltration and decreased vascular density in a mesothelioma xenotransplantation model [139].

New approaches for melanoma therapy combine measles virus targeted against melanoma-associated antigen (high molecular weight melanoma-associated antigen HMWMAA) and an insertion of the FCU1 gene (MV-FCU-1-α-HMWMAA) [140]. The latter encodes the yeast-derived prodrug convertases cytosine deaminase and uracil phosphoribosyltransferase [140]. The aim of the study was to enhance virus specificity for tumor cells in combination with an increased efficacy of chemotherapy by local prodrug conversion [140]. In a human melanoma xenograft model (A375M cells) tumors, that had reached a volume of 50 mm³, were treated intratumorally on five consecutive days with 1.44 × 10^5^ cell infectious units MV-FCU-1-α-HMWMAA followed by intraperitoneal administration of 5-fluorouracile twice daily on five consecutive days, starting three days after the last virus application [140]. This treatment scheme resulted in a significant growth retardation of treated tumors accompanied by a significantly prolonged survival time compared to controls [140].

A similar approach by using the same modification of the measles virus consisting of arming with a super cytosine deaminase (SCD), which is identical with the FCU-1 suicide gene, has been described in 2013 [141]. SCD is a fusion protein of yeast cytosine deaminase and uracil phosphoribosyltransferase and is able to convert the prodrug 5-fluorocytosine (5-FC) to 5-fluorouracil (5-FU) and afterwards to 5-fluorouridine-monophosphate [141]. In a human cholangiocarcinoma xenotransplantation study intratumoral infection with SCD-MV combined with a systemic 5-FC administration led to a reduction in tumor volume and a prolonged survival time [141]. Similar results have been documented for the application of SCD-armed MV in hepatocellular carcinoma cells *in vitro* and an *in vivo* xenotransplantation model in mice [142]. The preclinical safety of SCD-armed MV has been tested in a mouse and a rhesus macaque model [143]. A single intrahepatic application was well tolerated in both models, whereas daily repeated systemic applications with a simultaneous prodrug application resulted in adverse side effects, such as weight loss and hypothermia [143].

In murine models for B-cell lymphomas [144] and colon adenocarcinoma [145], treatment with a MV vaccine strain displaying an insertion of the granulocyte-macrophage colony-stimulating factor (GM-CSF) resulted in a retarded tumor progression and an increased median overall survival time [144,145]. In these studies, a total tumor regression was achieved in more than one-third of the animals treated with GM-CSF armed MV [145]. The oncolytic potential of this engineered MV has been attributed predominantly to the immune-modulatory effect of the transgene, leading to an increased adaptive immune response which amplifies the therapeutic efficacy of MV [145].

Another approach to increase MV activity was achieved by modifications to avoid antiviral counter-actions of the immune system. Although neoplastic cells often exhibit a limited antiviral response, the IFN-α and -β production may still be sufficient to inhibit intratumoral spread of oncolytic viruses [146,147]. To omit this virus spread-limiting factor of tumor cells, an oncolytic MV strain, based on EdMV, expressing the green fluorescent protein (eGFP; MV-eGFP-Pwt) was designed, which additionally expresses the P-gene of a MV wild-type strain [147]. This gene encodes for the P/V/C proteins which have the ability to antagonize IFN-α and -β induction and response [147]. Compared to the parental MV the modified MV-eGFP-Pwt shows markedly reduced capacity to induce IFN. However, compared to wild-type MV, the engineered virus expressing all the wild-type P, V, and C proteins was not able to prevent an IFN-α response completely in human Burkitt lymphoma cells (BJAB) and ARH-77 myeloma cells [147]. Intravenous administration of the modified MV in an *in vivo* model using immunocompromised mice with established human myeloma ARH-77 xenografts (0.3–0.5 cm in diameter) and MV-eGFP-Pwt (2 doses of 4 × 10^6^ TCID_50_) resulted in a significantly enhanced oncolytic potency compared to MV-eGFP [147].

In contrast to these studies, that were designated to limit the intratumoral antiviral immune response in oncolytic virus therapies, other investigations were performed with the goal to prime an antitumor immune response triggered by oncolytic virotherapy. Recently a study using MVeGFP was designed to elucidate the impact of an oncolytic virus-recruited antitumor immune response in a human melanoma *in vitro* model [109]. Hereby, MV caused an enhanced innate antitumor immune response. Additionally, a melanoma-specific adaptive immune response was triggered leading to melanoma cell death [109].

Apart from the discussed examples, many other approaches of engineered MV have been performed in the last years, several with promising effects, waiting for permission to be applied in clinical trials (Table 1).

**Table 1:** Examples of different measles viruses used for oncolysis including virus modification used for enhanced oncolytic activity/specificity, virus strain and studied tumors/tumor cell lines.

Virus modification		Virus strain	Tumor/tumor cell line	*in vitro*	*in vivo*	References
Measles virus without modification		Edmonston	human myeloma: ARH-77 cells, RPMI 8226 cells, JJN-3 cells, MM1 cells, KAS-6/1 cells, KMS-11 cells; primary myeloma cells;	x	x	[105]
		Edmonston	human ovarian carcinoma: SKOV3ip.1 cells; human fibrosarcoma: HT1080 cells; human epithelial lung carcinoma: A549 cells;	x	n.d.	[49]
		Edmonston Moraten	human ovarian carcinoma: OV202 cells, OV207 cells, SKOV3ip.1 cells;	x	x	[148]
		Edmonston-Zagreb	human T-cell lymphoma: SeAx cells, HUT 78 cells, MyLa cells; cutaneous T-cell lymphomas (CTCL);	x	x	[149]
		Schwarz	human mesothelioma: M11 cells, M13 cells, M31 cells, M47 cells, M56 cells, M61 cells;	x	n.d.	[110]
		Not detailed	human B-precursor acute lymphoblastic leukemia (ALL): 697 cells, Nalm-6 cells, SEM cells, REH cells; human Burkitt's lymphoma: Raji cells, Daudi cells; human T cell leukemia: Jurkat cells; primary chronic lymphocytic leukemia (CLL) and ALL cells;	x	x	[150]
		CAM-70; Schwarz MV wild-type: MV190112	human B cell lymphoma: BJAB cells; BJAB cells; marmoset B-lymphoblastoid: B95-8 cells; human Burkitt’s lymphoma: Akata cells, BL-41 cells, BL-41/cells, Daudi cells, Mutu cells, Jijoye cells, Namalwa cells, P3HR-1 cells, Raji cells, BLCL cells, LMP1-transduced: BJAB LMP1 cells;	x	n.d.	[151]
ß-galactosidase reporter gene (MVIacZ)		Edmonston	human lymphoma: DoHH2 cells, Raji cells;	x	x	[106]
CEA		Edmonston	human myeloma: RPMI 8226 cells; human fibrosarcoma: HT1080 cells;	n.d.	x	[135]
		Not detailed	human ovarian carcinoma: OV202 cells, OV207 cells, SKOV3ip.1 cells;	x	x	[148]
		Edmonston	human breast cancer: MDA-MB-231 cells, SkBr3 cells, MCF7 cells;	x	x	[152]
		Edmonston	human hepatocellular carcinoma: Hep-3B cells, HUH-7 cells;	x	x	[136]
		Not detailed	human histiocytic lymphoma: U-937 cells; human Burkitt's lymphoma: Raji cells; human myeloma: KAS-6/1 cells; human ovarian carcinoma: SKOV3ip.1 cells; human hepatocellular carcinoma: HUH-7 cells;	x	x	[153]
		Not detailed	human malignant glioma: U87 cells, U251 cells; primary glioblastoma multiforme: GBM12 cells;	x	x	[114]
		Edmonston-NSe	human breast cancer: MDA-MB-231 cells; human ovarian carcinoma: SKOV3ip.1 cells; human cerebellar medulloblastoma: TE671 cells;	x	n.d.	[154]
		Edmonston	human prostate cancer: PC-3 cells, DU-145 cells, LNCaP cells;	x	x	[155]
		Edmonston	human ovarian carcinoma: SKOV3ip.1 cells; human ovarian carcinoma;	x	Phase I clinical trial	[156]
		Edmonston	human hepatoblastoma (HB): Hep2G cells, HUH6 cells;	x	x	[24]
		Not detailed	human glioblastoma: GBM6 cells; primary human glioblastoma cells;	x	x	[157]
		Not detailed	human ovarian carcinoma: SKOV3ip.1 cells;	x	x	[158]
		Edmonston	human ovarian carcinoma: SKOV3ip.1 cells, IGROV1 cells; OV202 cells;	x	x	[159]
Single chain anti-body	CD38	Edmonston	human fibrosarcoma: HT1080 cells;	x	x	[121]
		Edmonston	human glioblastoma: U118 MGcells; human erythroleukemia: K562 cells; human Burkitt’s lymphoma: Raji cells, Ramos cells; human ovarian carcinoma: SKOV3ip.1 cells;	x	x	[115]
	CD20	Replicating MV	human fibrosarcoma: HT1080 cells;	x	x	[122]
	HER2/neu	Not detailed	human ovarian carcinoma: SKOV3ip.1 cells; human medulloblastoma: TE671 cells;	x	n.d.	[160]
	EGFRvIII	Edmonston	human glioblastoma: U118 MG cells; human erythroleukemia: K562 cells; human Burkitt’s lymphoma: Raji cells, Ramos cells; human ovarian carcinoma: SKOV3ip.1 cells;	x	x	[115]
		Edmonston-NSe	human glioblastoma: U118 cells; primary glioblastoma: GBM6 cells, GBM14 cells, GBM39 cells;	x	x	[161]
	PSMA	Edmonston	human prostate cancer: LNCaP cells, PC3 cells; human T cell leukemia: Jurkat cells; human Burkitt’s lymphoma: Raji cells; human multiple myeloma: KAS 6/1 cells	x	x	[162]
	CD133	Not detailed	Human fibrosarcoma: HT1080 cells; Human hepatocellular: HuH7 cells; Primary glioblastoma: NCH644 cells;	x	x	[51]
GM-CSF		Not detailed	human Burkitt’s lymphoma: Raji cells;	x	x	[144]
NIS		Edmonston	human ovarian carcinoma: SKOV3ip.1 cells, IGROV1 cells; OV202 cells;	x	x	[159]
		Edmonston	Human multiple myeloma: ARH 77 cells, KAS 6/1 cells, MM1 cells; primary myeloma cells;	x	x	[53]
		Not detailed	human glioblastoma: GBM6 cells; primary human glioblastoma cells;	x	x	[157]
		Edmonston	human multiple myeloma: KAS-6/1 cells;	n.d.	x	[163]
		Edmonston	human hepatocellular carcinoma: Hep-3B cells, HUH-7 cells;	x	x	[136]
		Edmonston	human pancreatic cancer: BxPC-3 cells, MiaPaCa-2 cells, Panc-1 cells;	x	x	[164]
		Edmonston	human prostate cancer: PC-3 cells, DU-145 cells, LNCaP cells;	x	x	[165]
		Not detailed	human multiple myeloma: MM1 cells, KAS-6/1 cells;	x	x	[166]
		Not detailed	human pancreatic cancer: BxPC-3 cells;	x	x	[167]
		Edmonston	human ovarian carcinoma: SKOV3ip.1 cells; human multiple myeloma: KAS6/1 cells;	x	x	[168]
		Edmonston	human malignant glioma: U87 cells, U251 cells; human primary glioblastoma: GBM6 cells, GBM10 cells, GBM12 cells, GBM39 cells, GBM43 cells, GBM44 cells;	x	x	[169]
		Edmonston	human head oral squamous cell carcinoma: SCC-25 cells, SCC-15 cells; anaplastic human thyroid carcinoma: SW579 cells; human hypopharyngeal carcinoma: FaDu cells;	x	x	[170]
		Edmonston	human medulloblastoma: D283med cells, UW426 cells;	x	x	[54]
		Edmonston	human head and neck cancer: HN3 cells, HN5 cells, PJ41 cells; human colorectal cancer: HCT116 cells;	x	x	[171]
NIS		Edmonston	human endometrial cancer: HEC-1-A cells, Ishikawa cells, KLE cells, RL95-2 cells, AN3CA cells; ARK-1 cells, ARK-2 cells, SPEC-2 cells;	x	x	[172]
		Edmonston B	human T-cell lymphoma: SeAx cells, MyLa2059 cells, HUT78 cells;	x	x	[173]
Human IL-13 at the C-terminus of the H-protein		Not detailed	human malignant glioma: U87, U118, U251 cells;	x	x	[174]
MMP		Edmonston B–based parental MV strain (NSe)	human fibrosarcoma: HT1080 cells; human glioblastoma: U87mg cells; human liver resection material with primary and secondary tumors;	x	n.d.	[128]
		Not detailed	human fibrosarcoma: HT1080 cells;	x	x	[125]
αVβ3-integrin targeted (RGD or echistatin domains)		Edmonston	multiple myeloma xenografts;	x	x	[175]
		Not detailed	human multiple myeloma: KAS6/1 cells;	x	x	[176]
Human light immunoglobulin chain reporter gene		Edmonston	Human multiple myeloma: ARH-77 cells, KAS 6/1 cells; human T cell leukemia: Jurkat cells;	x	x	[177]
NAP		Edmonston	human breast cancer: MCF-7 cells, MDA-MB-231 cells;	x	x	[178]
Suicide gene SCD/FCU-1		Not detailed	human ovarian carcinoma: OAW42 cells, SKOV3 cells; primary human ovarian carcinoma cells;	x	n.d.	[179]
		Edmonston B	human melanoma: A375M, Mel888, pMelL, and SK-MEL-28 cells;	x	x	[140]
		Schwarz	human cholangiocarcinoma: RBE, HuCCT1, TFK-1 cells;	x	x	[141]
		Not detailed	Primary murine and rhesus macaque hepatocytes;	x	x	[143]
		Mérieux	Human cholangiocarcinoma: HuCCT-1 cells; Human hepatocellular carcinoma: Hep3B cells; Human colorectal adenocarcinoma: HCT116 and HCT15 cells;	x	n.d.	[180]
DARPins	EGFR	Edmonston	human adenocarcinoma: AU565 cells, SK-Br-3; human breast ductal carcinoma: BT-474 cells, MCF-7 cells; human colon adenocarcinoma: Caco-2 cells, HT-29 cells, SW-620; human fibrosarcoma: HT1080 cells; human ovarian carcinoma: SK-OV-3 cells; human malignant glioma: U87mg cells;	x	x	[181]
	Her2/neu					
	EpCAM					
MicroRNA-sensitive (containing target sites for microRNA-7 in the 3' untranslated region of the viral fusion gene)		Edmonston-B vaccine lineage	human malignant glioma: U87 cells; primary human brain tissue from the peripheral invasion front of a glioma resection;	x	x	[134]

**CD**, cluster of differentiation; **DARPins**, designed ankyrin repeat proteins; **EGFR**, epidermal growth factor receptor; **EpCAM**, epithelial cell adhesion molecule; **GM-CSF**, granulocyte macrophage colony stimulating factor; **HER2/neu**, human epidermal growth factor receptor 2; **IL**, interleukin; **MMP**, matrix metalloproteinase; **MV**, measles virus; **MV-H/hEGF**, measles virus H glycoprotein-human epidermal growth factor hybridprotein; **n.d.**, not done; **NIS**, human thyroidal sodium iodide symporter; **PSMA**, prostate-specific membrane antigen; **SCD**, super cytosine deaminase; **not detailed**, used measles virus strain was not further specified in the Materials and Methods section of the publication.

### 3.3. Clinical Trials with Oncolytic Measles Virus

2005, Heinzerling *et al.* [149] published an open-label dose escalation phase I clinical trial involving five patients suffering from a cutaneous T-cell lymphoma (CTCL). Live attenuated Edmonston-Zagreb vaccine strain was administered intratumorally at a total of 16 injections (ranging from 10²–10³ TCID_50_) accompanied by IFN-α injection two days before and after virus application [149]. Interferon-α was given to restrict MV replication to IFN-resistant CTCL-cells and thus enhance the safety profile of the MV-based therapy. Tumor regressions were seen in 5 out of 6 lesions (one patient with two treated tumors) within a month after treatment initiation [149]. Interestingly, several distant tumor localizations without an intratumoral infection also regressed or displayed growth retardation [149]. Subsequent analysis of biopsies of treated tumors revealed the presence of MV nucleoprotein within large tumor cell syncytia [149]. Moreover, a distinct local immune reaction was observed characterized by an intratumoral increase of CD8^+^ T-cells, an increased number of IFN-γ mRNA and a reduced number of CD4 mRNA transcripts compared to biopsies of the same lesions before treatment [149]. Interestingly, an augmentation of humoral anti-measles antibody titers was seen in all cases [149].

The first clinical trial using an engineered MV expressing the human carcinoembryonic antigen (CEA, MV-CEA) antigen was published by Galanis *et al.* in 2010 [156]. In this trial, 21 patients with recurrent Taxol- and platinum-refractory ovarian cancer and CEA levels within the reference range, were treated with up to six-fold intraperitoneal administration of MV-CEA, every four weeks, at seven different dose levels ranging from to 10³ to 10^9^ TCID_50_ [156]. The treatment improved the median survival time of the patients from expected six months (as recorded from historic controls) to 12.15 months by good tolerance and no observed dose-limiting toxicity, treatment-induced immunosuppression, development of anti-CEA antibodies, increase in antibody titers, or virus shedding in urine or saliva [156].

Until now a number of additional, often ongoing, phase 1 and 2 clinical studies applying modified-oncolytic MV have been conducted [45,182]. Two studies use engineered EdMV strains expressing the human CEA and the human thyroidal sodium iodide symporter (NIS, MV-NIS) respectively [45]. Intraperitoneal administration of MV-CEA is used in patients with recurrent ovarian cancer and leads to a dose-dependent stabilization of the disease in all patients of the higher dose levels (10^7^ to 10^9^ TCID_50_) compared to only 5 of 12 patients treated in the lower dose range (10^3^ to 10^6^ TCID_50_). At all dose levels mentioned, only few adverse side effects are noted [45]. A third clinical trial comprises intratumoral and resection cavity administration of MV-CEA in patients with recurrent *glioblastoma multiforme*. In this attempt intratumoral virus application precedes operation by three days followed by resection cavity administration of MV-CEA after surgery [45]. A fourth clinical trial includes intravenous administration of MV-NIS with or without cyclophosphamide treatment in patients with multiple myeloma [45]. Only recently, a further clinical trial applying MV-NIS to two patients with myeloma and plasmacytoma, respectively, provided promising results [183].

## 4. Canine Distemper Virus as an Oncolytic Virus

Canine distemper virus shares many similarities with human measles virus [184] and, therefore, represents an interesting candidate for viral oncolysis. Though measles virus represents an intensively studied candidate virus for tumor therapy as outlined before, actually, there are only few published studies regarding the oncolytic potential of canine distemper virus. Despite numerous *in vitro* investigations about the impact of CDV infection on cell lines initially originating from tumor tissue, the majority of studies investigated infection related pathogenesis without paying any attention to the potential antitumor activity of this canine morbillivirus.

Whereas the attenuation of measles virus vaccine strains can be attributed to genetic modifications in almost every viral protein affecting host cell tropism and the ability to inhibit cellular antiviral defense mechanisms [185], the basis of attenuation of the CDV vaccine strains is largely unknown. Recently, Dietzel *et al.* (2011) reported that the modifications of the CDV matrix protein could be one factor influencing the virulence by affecting particle composition and envelope protein distribution in polarized epithelial cells [186].

CDV infection of a human cervical tumor derived cell line (HeLa cells) results in apoptosis of the tumor cells, triggered by the intrinsic pathway, as demonstrated by an increase of the amount of the cleaved active form of the caspase-3 protein [187]. Similarly, enhanced rates of apoptosis after CDV infection have been described in canine lymphoid cell lines (CLGL-90 chronic large granular lymphocytic T cell leukemia cells and 17–71 acute B cell lymphoma cells) [89]. In these lymphoid tumor cell lines, as well as in isolated tumor cells from dogs with spontaneous malignant B and T cell lymphoma and non-neoplastic canine blood mononuclear cells, CD150 (SLAM) mRNA has been detected, indicating that CD150 (SLAM) renders these cells susceptible for CDV infection [89]. Surprisingly, CD46 mRNA, another important morbillivirus receptor often used by attenuated strains, has been detected only in neoplastic B and T lymphocytes while non-neoplastic cells and lymphoid cell lines remained negative [89]. In addition to lymphoid cell lines, canine distemper virus is able to infect canine histiocytic sarcoma cell lines (CCT cells and DH82 cells) [188,189] and induces apoptosis in CCT cells [189]. A CDV infection of DH82 cells leads to an increased expression of proinflammatory cytokines, namely IL-1, IL-6, and tumor necrosis factor (TNF) [190]. Conceivably, production of these cytokines may result in an inflammatory microenvironment enhancing the Th1 polarizing capacities as described for human mesothelioma cells co-cultured with human dendritic cells and infected with MV [110]. In the latter study, incubation of MV-infected, apoptotic tumor cells with dendritic cells leads to phagocytosis of the apoptotic cells accompanied by abrupt dendritic cell maturation [110]. These dendritic cells produce an increased amount of proinflammatory cytokines, namely IL-1ß, IL-6, TNF, and IFN-α [110]. The produced cytokines enhance the immunogenicity of MV-infected tumor cells and creates an inflammatory environment with Th1 polarizing capacities [110].

Another hallmark of many malignant tumors represents their ability to grow invasively and metastasize to other organs. Therefore, tumors have to modulate the extracellular matrix, often represented by an imbalance in the expression of matrix metalloproteinases (MMPs) and their inhibitors, for example tissue inhibitors of matrix metalloproteinases (TIMPs) and the reversion-inducing cysteine-rich protein with Kazal motifs (RECK) [51,191,192,193,194]. In CDV-infected DH82 cells a down‑regulation of MMP-2, TIMP-1, and TIMP-2 has been observed while RECK displays a higher number of mRNA transcripts [188]. These results suggest that a CDV infection of canine histiocytic sarcoma cells restores RECK expression and might positively influence the tumor behavior and reduce its malignant potential [188]. The lesser aggressive phenotype is assumed due to respective findings in human medicine where tumors with a normal or high RECK expression are often associated with a better prognosis, whereas tumors lacking a RECK expression or displaying low amounts of this protein are commonly related to a poorer prognosis [192,195,196,197]. 

## 5. Summary and Future Perspectives

This review highlights recent findings and provides new insights into different strategies in tumor treatment using morbilliviruses with special emphasis of virus modifications to enhance tumor cell sensitivity, therapeutic efficacy, and to reduce pathogen associated adverse side effects. MV was one of the first viruses leading to tumor regression after vaccination or accidental infection. This resulted in numerous studies investigating the potential benefit of a MV infection for tumor therapy especially in neoplasms, which are difficult to treat. *In vitro*, many different modifications of the MV were performed in order to improve tumor cell specificity and achieve an enhanced anti-cancer immune response or to regulate the tumor microenvironment, namely vascularization. Unfortunately, only a limited number of clinical trials have been performed until now.

Despite many similarities between CDV and MV, both viruses differ significantly in their potential to induce neuropathological lesions. Whereas, often encountered in CDV, such lesions are reported only occasionally in the course of MV-infections, often depending on the respective virus strain. MV representing a strictly human pathogen is further opposed to CDV infecting a much broader host range including many carnivore species. Nevertheless, the above noted similarities between MV and CDV allow an extrapolation of possible treatment schemes to veterinary medicine and render the canine pathogen an interesting, translational model for various human tumors. 

Furthermore, certain canine tumors frequently reflect the situation in human cancers. This can be attributed to the same environment including for example exposure to the same carcinogens. Moreover, similarities in tumorigenesis, tumor morphology, prognosis, and diagnosis have been noticed in certain human and canine neoplasms. Additionally, many treatment schemes of canine neoplasms are similar to their human counterparts, underlining the potential usefulness of translational studies with benefits for both, human and veterinary medicine. 

Currently, the mode of action of oncolytic morbilliviruses is still undetermined in many cases and recognized mechanisms refer frequently to *in vitro* systems only. Therefore, detailed *in vivo* studies are highly requested for a better understanding of the complex interaction between oncolytic viruses and tumor cells as well as their impact on the microenvironment and anti-tumor immune reactions.

Conclusively, several recent findings and promising studies substantially contributed to a better understanding of the complex interaction between oncolytic viruses and different tumor types. These studies raised the hope for developing new therapeutic options in tumors difficult to treat so far by using viral oncolysis as a new treatment strategy. However, despite promising results, further studies are still needed to unravel the complex and only partially understood underlying mechanisms.
